# A 7-Hydroxybenzoxazinone-Containing Fluorescence Turn-On Probe for Biothiols and Its Bioimaging Applications

**DOI:** 10.3390/molecules24173102

**Published:** 2019-08-27

**Authors:** Bin Li, Datong Zhang, Ruibing An, Yaling Zhu

**Affiliations:** School of Chemistry and Pharmaceutical Engineering, Qilu University of Technology (Shandong Academy of Sciences), 3501 Daxue Road, Jinan 250353, China

**Keywords:** fluorescence probe, biothiols, 7-hydroxy-3-phenyl-benzoxazinone, large Stokes shift, 2,4-dinitrobenzenesulfonate

## Abstract

In this work, a novel 7-hydroxybenzoxazinone-based fluorescent probe (**PBD**) for the selective sensing of biothiols is reported. Upon treatment with biothiols, **PBD** shows a strong fluorescence enhancement (up to 70-fold) and a large Stokes shift (155 nm). Meanwhile, this probe exhibits high resistance to interference from other amino acids and competing species. **PBD** features good linearity ranges with a low detection limit of 14.5 nM for glutathione (GSH), 17.5 nM for cysteine (Cys), and 80.0 nM for homocysteine (Hcy), respectively. Finally, the potential utility of this probe for biothiol sensing in living HeLa cells is demonstrated.

## 1. Introduction

Sulfhydryl-containing amino acids, such as cysteine (Cys), glutathione (GSH), and homocysteine (Hcy), play key roles in maintaining appropriate physiological and biological processes [1,2,3,4,5]. Malfunction of Cys levels has been associated with hair depigmentation, slowed growth rate, liver damage, edema, and so on [6]. Elevated levels of Hcy result in cardiovascular and Alzheimer’s disease [7,8]. GSH is the most abundant intracellular non-protein thiol and its concentration is within millimolar range. GSH deficiency is linked with cancer, liver damage, and neurodegenerative disease [9,10,11]. Owing to their significant roles, it is of great importance to develop rapid, simple and reliable methods for monitoring the levels of biothiols in biological systems.

Among various detection methods, fluorescent-probe-based detection has been accepted as one of the most efficient methods due to its advantages of having operational simplicity, being low cost, working under in situ real time conditions and involving no invasive bioimaging [12,13,14,15,16].

**Scheme 1:**
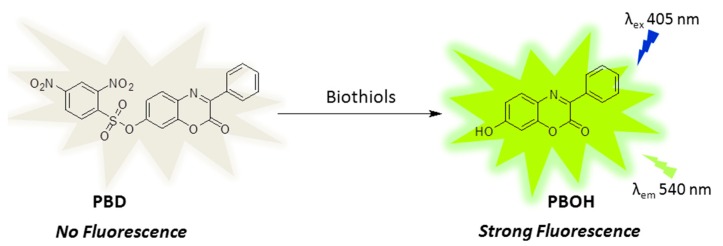
Design diagram of fluorescent probe **PBD** (2,4-dintrobenzenesulfonate of 7-hydroxy-3-phenylbenzoxazinone) for biothiol detection.

Great efforts have been directed to the development of fluorescent probes toward biothiols (Cys, Hcy, and GSH) [17,18,19,20,21,22,23]. Many probes have been constructed based on traditional fluorescent dyes, such as fluorescein [24], naphthalimide [25], coumarin [26], cyanin [27], BODIPY (boron-dipyrromethene) [23], xanthene [28], 2-(2′-hydroxyphenyl)benzothiazole [29], and dicyanomethylene-4*H*-pyran derivatives [30]. In spite of some sensors with good sensing performance, many of these probes still suffer from poor solubility, low sensitivity, low quantum yield, and a time-consuming detection process. Thus, it would be beneficial to develop fluorophores with a new structural skeleton. Up until now, fluorescent dyes with a 7-hydroxybenzoxazinone skeleton have received little attention [31,32,33]. We prepared 7-hydroxy-3-phenylbenzoxazinone (**PBOH**) and evaluated its optical property. **PBOH** exhibits strong yellow-green fluorescence and a large Stokes shift (155 nm). We envisioned that masking the hydroxyl group in **PBOH** with a 2,4-dinitrophenylsulfonyl group (DNBS) could generate a new fluorescent probe (**PBD**) for the selective detection of biothiols (Scheme 1). As they are well-known, arenesulfonates and arenesulphonamides can be readily cleaved by thiolate anions, and, therefore, the 2,4-dintrobenzenesulfonyl (DNBS) group has often been chosen as the trigger group for thiols [24,34,35,36,37,38]. More importantly, the DNBS group can totally quench fluorescence, thus reducing background interference, because of its strong electron-withdrawing ability. We speculate that biothiols induce cleavage of the ester bond in the DNBS-PBOH conjugates, which can release free **PBOH** and thereby restore the strong emission, thus realizing a selective fluorescence off-on recognition of biothiols. The new probe **PBD** indeed exhibits a significant off-on response to biothiols. The investigation of **PBD** suggested that it possesses a strong anti-interference ability while sensing biothiols, with a low detection limit of 14.5 nM for GSH, 17.5 nM for Cys, and 80.0 nM for Hcy, respectively. In addition, **PBD** was successfully applied for biothiol detection in living HeLa cells.

## 2. Results and Discussion

**PBOH** showed strong fluorescence (Φ = 0.60) with a maximum at 540 nm and an absorption band centered at 385 nm in EtOH-PBS buffer (Phosphate Buffered Saline) solution (10 mM, pH = 7.4, 4:6, *v/v*) (Stokes shift = 155 nm). Owing to the quenching effect of the DNBS group, **PBD** alone exhibited extremely weak fluorescence with a low quantum yield at 540 nm (Φ = 0.011), and its absorption maximum was blue-shifted to 340 nm under the same aqueous conditions (Figure S1). As expected, upon addition of biothiols, a remarkable fluorescence intensity enhancement at 540 nm was observed. As such, we constructed a turn-on fluorescent probe for biothiols.

### 2.1. Time-Dependence and pH Effect

**Figure 1:**
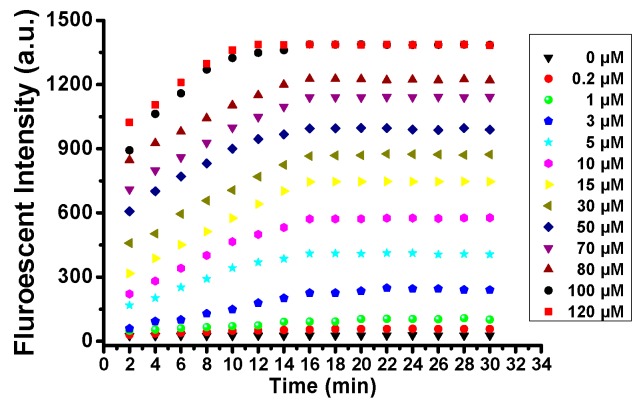
Time-dependent course of the interaction of **PBD** (10 µM) with GSH in EtOH-PBS buffer (Phosphate Buffered Saline) (10 mM, pH 7.4, 4:6, *v*/*v*). Fluorescence intensity was recorded at 540 nm with λ_ex_ = 405 nm.

The sensory effect of **PBD** is exemplified by its reaction with GSH. The emission intensity was recorded at different time points to evaluate the response time between **PBD** and various amounts of GSH. As shown in Figure 1, after addition of GSH, the fluorescence emission peak at 540 nm reached a plateau in about 16 min and maintained stability for a long time.

Kinetic analysis was conducted next. Ten equivalents of GSH, Cys, and Hcy were added into the solutions of **PBD** (10 µM), respectively. Through monitoring the fluorescence emission at 540 nm, the reactions were found to obey a typical pseudo-first-order and the rate constants were determined to be 0.25 min^−1^, 0.26 min^−1^, and 0.20 min^−1^ for GSH, Cys, and Hcy respectively (Figure S5).

Furthermore, we evaluated the pH effect on the sensory response of **PBD** (Figure S3). **PBD** itself was inert to pH change, indicative of the excellent stability of the probe over a broad pH range. By contrast, the probe exhibited a marked fluorescence response to GSH within the pH range 7 to 9, thus enabling detection of biothiols across a relatively wide pH range.

The following experiments of **PBD** were performed in EtOH-PBS buffer (10 mM, pH 7.4, 4:6, *v*/*v*) and 405 nm was chosen as the excitation wavelength in the fluorescent measurements. For convenience, the fluorescent intensity was recorded at 16 min after the addition of GSH.

### 2.2. Sensitivity and Selectivity Studies

**Figure 2:**
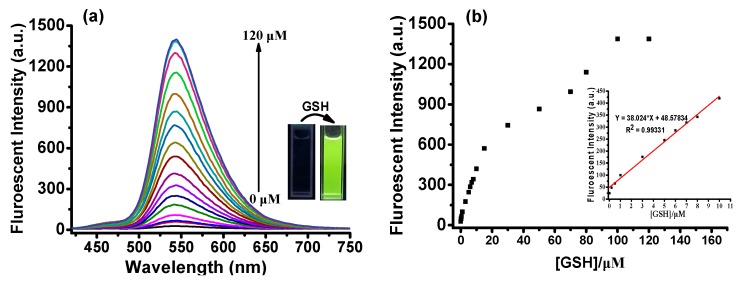
(**a**) Fluorescence response of **PBD** (10 μM) upon the addition of GSH (0–120 μM) in EtOH-PBS buffer (10 mM, pH 7.4, 4:6, *v*/*v*). Inset: Fluorescence images of **PBD** (10 μM) in the absence (left) and presence (right) of GSH under a 365 nm UV lamp. (**b**) Fluorescence intensity of **PDB** (10 μM) at 540 nm as a function of GSH concentration (0–120 μM) in EtOH-PBS buffer (10 mM, pH 7.4, 4:6, *v:v*). Inset: the linear relationship between fluorescence intensity and GSH at low concentrations.

Concentration-dependent tests of **PBD** with GSH showed that the fluorescence intensity at 540 nm increased gradually with increasing GSH amounts until a stable signal was reached at 100 µM (Figure 2). The fluorescence enhancement could be about 70-fold when 10 equivalents of GSH were added to the solution of **PBD**. As shown in Figure 2, upon treatment with GSH, the testing solution gave rise to a strong yellow-green fluorescence which is consistent with the emission spectrum of dye **PBOH**. A calibration plot of the signal to the GSH concentrations from 0 µM to 10 µM showed good linearity (R^2^ = 0.9933), indicating that **PBD** can quantitatively detect GSH within this range. The detection limit of **PBD** toward GSH was found to be 14.5 nM based on the 3σ/slope method [39,40], thus enabling a highly sensitive detection of GSH. We also evaluated the response effect of Cys and Hcy to **PBD**. Under the same conditions, **PBD** exhibited similar optical behavior to Cys and Hcy with the detection limits being 17.5 nM and 80.0 nM, respectively (Figure S4).

**Figure 3:**
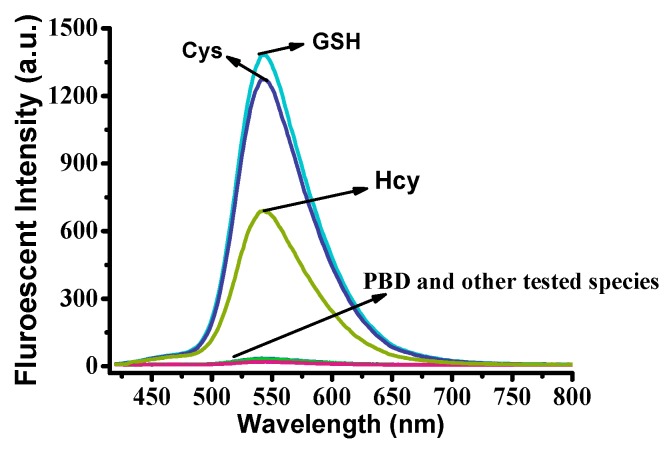
Fluorescence spectra of **PBD** (10 µM) with biothiols (100 µM) and various tested analytes (200 µM). λ_ex_ = 405 nm.

**Figure 4:**
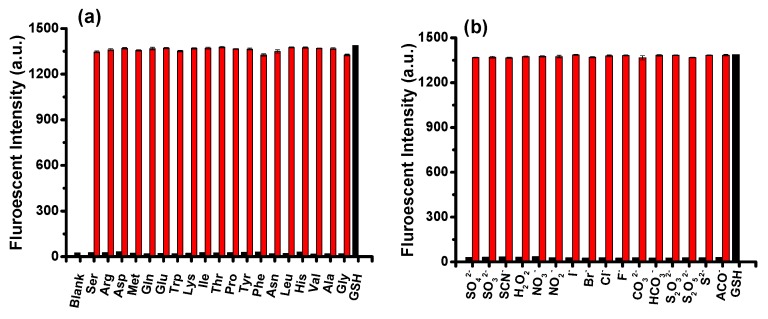
(**a**,**b**) Selectivity and competition of **PBD** (10 µM) to GSH (100 µM) and other analytes (200 μM). Black bars represent the fluorescence intensity (540 nm) of a single analyte with probe **PBD**. Red bars represent the fluorescence intensity (540 nm) of **PBD** to GSH in the presence of various tested analytes. λ_ex_ = 405 nm.

The selectivity of **PBD** for biothiols (100 μM) was evaluated by screening its response to potential competing species (200 μM), including various natural amino acids (Val, Glu, Leu, Met, Asn, Ala, His, Trp, Phe, Ser, Arg, Lys, Gln, Asp, Ile, Thr, Tyr, Pro, and Gly), sulfur species (Na_2_S, KSCN, Na_2_SO_3_, Na_2_SO_4_, Na_2_S_2_O_3_, and Na_2_S_2_O_4_), oxygen species (H_2_O_2_), and common salts (KF, NaCl, KBr, KI, Na_2_CO_3_, NaHCO_3,_ NaNO_2_, NaNO_3_, and AcONa). Figure 3 illustrates that treatment of **PBD** solution with 10 equivalents of GSH resulted in a 70-fold enhancement at 540 nm. Cys gave rise to a similar fluorescence increase. Hcy induced an increment half that of GSH. High concentrations (200 µM) of interfering species induced negligible changes. Following on from this, competition studies were investigated by treating **PBD** with GSH (100 μM) in the presence of 20 equivalents of other analytes. As shown in Figure 4a,b, addition of GSH to the probe solution resulted in a robust signal enhancement in spite of the coexistence of competing species, demonstrating that probe **PBD** displays strong immunity to interference while sensing biothiols.

### 2.3. Sensing Mechanism Study

**Scheme 2:**
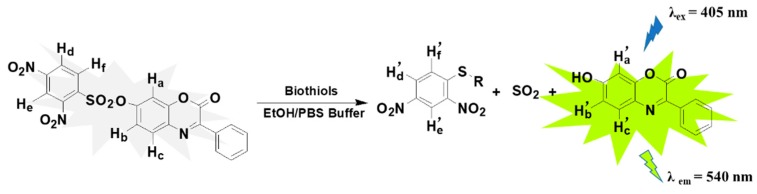
Schematic sensing mechanism of **PBD** toward biothiols.

The fluorescence turn-on response of **PBD** to biothiols may be attributed to a thiol-anion-mediated S_N_Ar process which led to the cleavage of 2,4-dinitrophenylsulfonate (Scheme 2). A comparison of the fluorescence spectral profiles of the reaction solution and the precursor compound provides evidence for the proposed mechanism. Upon addition of GSH to non-fluorescent **PBD,** a turn-on fluorescent signal at 540 nm was observed and the emission intensity nearly reached the intensity of **PBOH**. In addition, we successfully isolated the fluorescent product of the reaction by column chromatography. As expected, the ^1^H-NMR spectrum of the isolated fluorescent product is consistent with that of **PBOH**. Thus, the above results confirmed that dye **PBOH** was released via the cleavage of sulfonate in **PBD** mediated by GSH.

**Figure 5:**
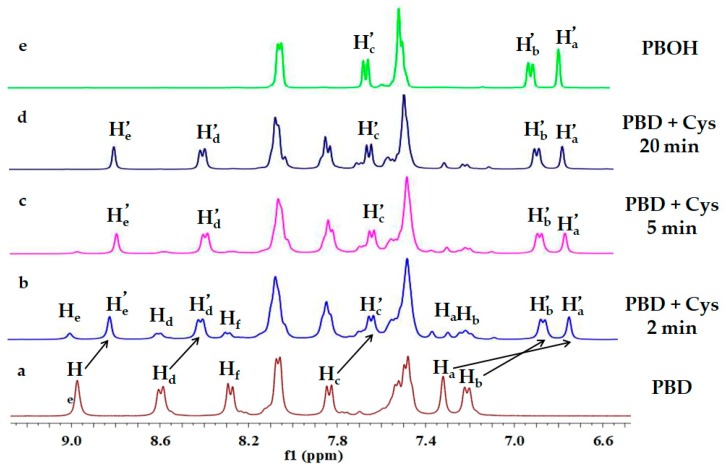
Partial ^1^H-NMR spectra of **PBD** before the addition (**a**) and the change after addition of Cys, 2 min (**b**), 5 min (**c**), and 20 min (**d**), and of **PBOH** (**e**) in DMSO-*d*_6_/D_2_O (8/2, *v/v*).

To confirm the sensing mechanism, we also carried out ^1^H-NMR titration experiments to track product formation. After the addition of excessive Cys to **PBD** in DMSO*-d_6_*/D_2_O (8/2, *v/v*), the signals of **PBD** (H_a_ and H_b_) gradually declined. At the same time, three peaks corresponding to the protons (H_a_’, H_b_’, and H_c_’) attributable to **PBOH** began to appear, indicating that **PBOH** had been released. With the continuation of this chemical reaction, the signals of **PBOH** (H_a_’, H_b_’, and H_c_’) gradually increased. The signals of **PBD** (H_a_ and H_b_) had almost completely disappeared at 20 min. These results demonstrate the cleavage of the DNBS group and the production of fluorophore **PBOH** (Figure 5).

**Figure 6:**
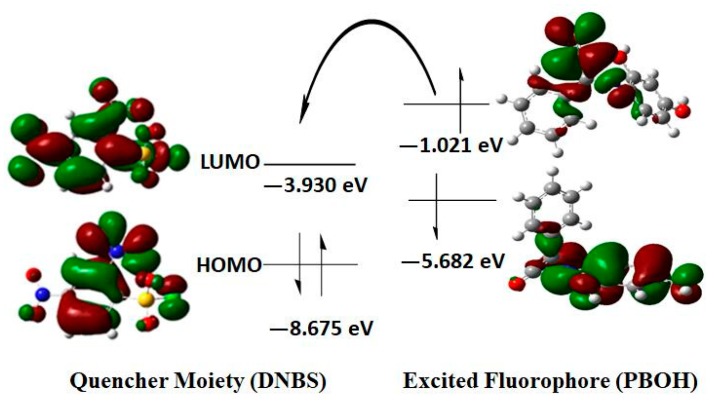
Frontier orbital diagrams of **PBOH** and 2,4-dinitrophenylsulfonyl (DNBS). Orbital energies were calculated using Gaussian 03 program at B3LYP/6-31 g (d, p) level.

To elucidate further the *off-on* sensing mechanism of probe **PBD** towards biothiols, DFT (Density functional theory) calculations were performed. The frontier orbital diagram indicates that the LUMO energy level of DNBS (−3.930 eV) is much lower than that of fluorophore the **PBOH** (−1.021 eV) (Figure 6), which implied that the photo-induced electron transfer (PET) process can happen from the fluorophore moiety to the DNBS group. Owing to the effective fluorescence quenching effect of DNBS, **PBD** is essentially nonfluorescent. After removal of the DNBS group, the fluorescence is recovered.

### 2.4. Live Cell Imaging

**Figure 7:**
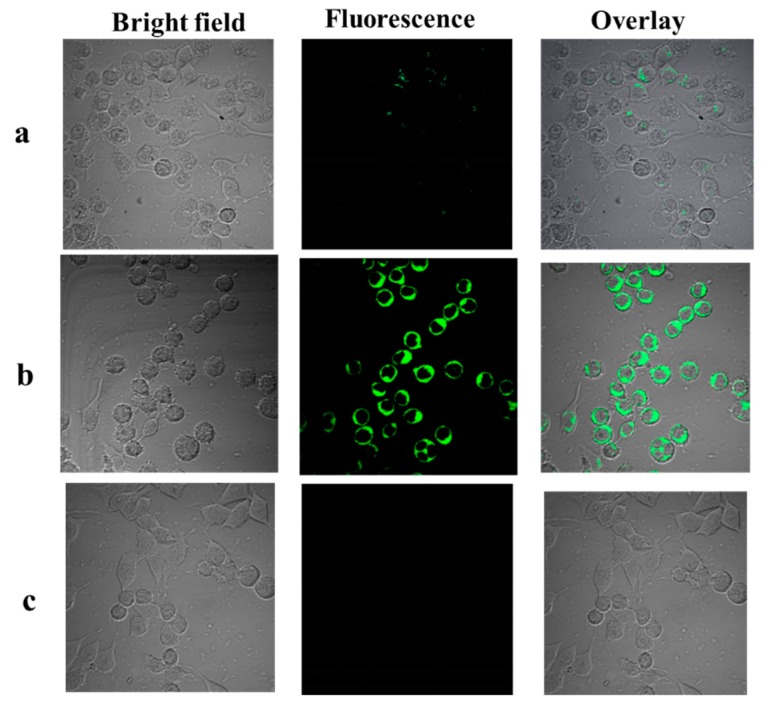
Confocal fluorescence images of HeLa cells. (**Left**) bright-field image; (**middle**) fluorescence image; and (**right**) overlayed image. (**a**) Cells treated with *N*-ethylmaleimide (NEM) (1 mM) for 30 min followed by incubation with **PBD** (10 μM) for 20 min. (**b**) Cells incubated with **PBD** (10 μM) for 20 min at 37 °C. (**c**) Untreated cells.

Having demonstrated its excellent responsiveness and anti-interference in sensing biothiols, we next examined whether **PBD** can be used to detect intracellular thiols in living cells. As shown in Figure 7, after incubating HeLa cells with **PBD** for 20 min, strong yellow-green fluorescence could be observed inside the cells under excitation at 405 nm. In the control experiments, cells were pretreated with *N*-ethylmaleimide (NEM) for 30 min to remove free thiols, and subsequently incubated with **PBD** for another 20 min. After being washed with PBS buffer three times, the cells showed almost no fluorescence under a confocal fluorescence microscope. The results showed that **PBD** is cell-permeable and can sense intracellular biothiols in living cells.

## 3. Materials and Methods

### 3.1. Materials.

All chemicals used were of analytical grade and were obtained from commercial suppliers. TLC (Thin-layer chromatography) silica gel plates (GF-254) and silica gel (200-300 mesh) for column chromatography were obtained from Qingdao Marine Chemicals, China. Deionized water was employed throughout all experiments. ^1^H-NMRand ^13^C-NMRspectra were acquired on a Bruker AVANCE II 400 spectrometer (Bruker, Switzerland), respectively. HRMS (High Resolution Mass Spectrometry) spectra were measured with a 6510-Q-TOF spectrometer (Agilent, USA). UV–Visible absorption spectra were recorded by a TU-1901 spectrometer (Beijing, China). Fluorescent measurements were performed on a Lengguang F97Pro FL Spectrophotometer (Shanghai, China). The pH measurement was carried out on a Leici PHS-3C pH meter (Shanghai, China). All spectra were recorded at room temperature.

### 3.2. Synthesis and Characterization of Compound Information

The four-step synthetic route is outlined in Scheme 3. The details of the synthesis and structural characterization are shown in the Supporting Information (Figures S8–S14).

## 4. Conclusions

In sum, we have developed a new off-on fluorescent probe **PBD** for biothiols by modifying a fluorescent dye 7-hydroxy-3-phenyl-benzoxazinone with a DNBS unit as a biothiol-specific recognition moiety. In response to biothiols, **PBD** displays significant fluorescence enhancement (up to 70-fold) and a large Stokes shift (155 nm). The probe exhibits a low detection limit as well as excellent selectivity and anti-interference ability toward biothiols over competing species. Finally, this probe has been used to sense biothiols in live HeLa cells.

## Figures and Tables

**Scheme 3:**
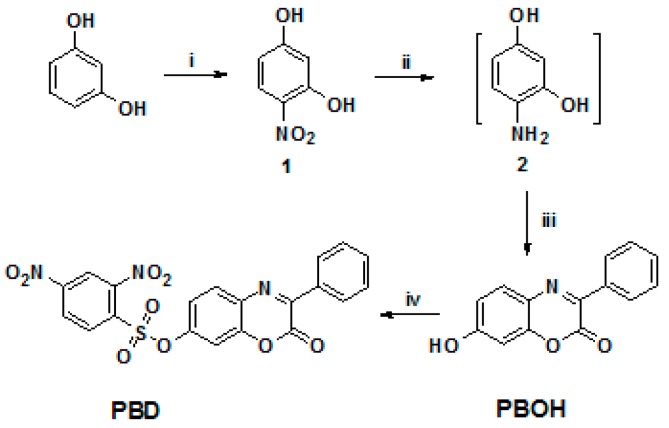
Synthesis of **PBD**. Reagents and conditions: (**i**) fuming HNO_3_, CHCl_3_, and CH_3_COOH, r.t.; (**ii**) Pd/C, H_2_, and CH_3_COOCH_2_CH_3_, r.t.; (**iii**) PhCOCOOH, EtOH, and CH_3_COOH, r.t; (**iv**) 2,4-Dinitrobenzenesulfonyl chloride, Et_3_N, and THF, r.t.

## Supplementary Materials

The supplementary materials are available online.
