# Supplementary material for: A 7-Hydroxybenzoxazinone-Containing Fluorescence Turn-On Probe for Biothiols and Its Bioimaging Applications
Source: Molecules. 2019 Aug 27;24(17):3102. doi: 10.3390/molecules24173102 (PMC6749190; doi:10.3390/molecules24173102)
Supplement: Supplementary file 1 [file molecules-24-03102-s001.pdf]

Supporting Information

A 7-hydroxybenzoxazinone-Containing Fluorescence Turn-On Probe for Biothiols and Its Bioimaging Applications

Bin Li, Datong Zhang*, Ruibing An, Yaling Zhu

School of Chemistry and Pharmaceutical Engineering, Qilu University of Technology (Shandong Academy of Sciences),3501 Daxue Road, Jinan 250353, P. R. China.

E-mail: dtzhang@qlu.edu.cn

*For correspondence

Synthesis and characterization of compounds .... - 2 -

UV-Vis absorption spectroscopy and fluorescence spectra .... - 3 -

Time-dependent fluorescence intensity of PBD with Cys and Hcy .... - 3 -

Quantum yield calculation .... - 4 -

Effect of pH on fluorescence detection of biothiols .... - 4 -

Calculation of detection limit .... - 4 -

Dynamic studies of PBD with biothiols .... - 5 -

Fluorescence response of PBOH, PBD with GSH and PBD .... - 6 -

Cytotoxicity measurements of PBD .... - 6 -

NMR and HRMS data .... - 7 -

References .... - 10 -

Synthesis and characterization of compounds

Synthesis of compound 1 [1]. To a solution of resorcinol (3.0 g, 27.27 mmol) in a mixture (2:1, 150 mL) of chloroform and acetic acid was slowly added a solution of nitric acid (2.0 mL) in acetic acid (45 mL). After being stirred for 2 h, the reaction mixture was quenched with water (50 mL) and extracted with CH_2Cl_2. The organic layer was dried over Na_2SO_4 and concentrated under reduced pressure. The residue was purified by flash column chromatography on silica gel (EtOAc/petroleum ether, *v*/*v*, 1:4) to give the desired product as a yellow solid (750 mg, 25% yield): ^{1}H–NMR (400 MHz, DMSO-d_6) δ 10.97 (s, 1H), 10.82 (s, 1H), 7.89 (d, *J* = 9.1 Hz, 1H), 6.38–6.42 (m, 2H).

Synthesis of compound PBOH. Compound 1 (200 mg, 1.29 mmol) was dissolved in 20 mL EtOH and palladium 20% on activated carbon (75 mg) was added. The reaction mixture was stirred at room temperature for 12 h under hydrogen atmosphere. The reaction mixture was filtered and the filtrate was concentrated. 162 mg of compound 2 was obtained as a brown solid and used directly for the next step without further purification. Compound 2 (150 mg, 1.2 mmol) was dissolved in 10 mL ethanol. After the addition of benzoylformic acid (180 mg, 1.2 mmol) and 0.7 mL acetic acid, the mixture was stirred at room temperature for 4 h and concentrated in vacuo. The residue was chromatographed on silica gel with ethyl acetate/petroleum ether (*v*/*v*, 3:1) to afford 163 mg **PBOH** as a yellow powder and the overall yields of two steps were 44%. ^{1}H–NMR (400 MHz, DMSO-d_6) δ 10.69 (s, 1H), 8.13 (d, *J* = 6.4 Hz, 2H), 7.66 (d, *J* = 8.8 Hz, 1H), 7.50–7.48 (m, 3H), 6.86 (d, *J* = 8.8 Hz, 1H), 6.75 (s, 1H). HRMS (ESI) calcd for $[M+H]^+$ $C_{14}H_{10}NO_3$: 240.0661, found: 240.0669.

Synthesis of compound PBD. A solution of compound **PBOH** (60 mg, 0.25 mmol), 2,4-dinitrobenzenesulfonyl chloride (73 mg, 0.27 mmol) and triethylamine (69 μL, 0.48 mmol) in anhydrous tetrahydrofuran (6 mL) was stirred at room temperature for 2.5 h under nitrogen atmosphere. The solvent was removed under reduced pressure. The crude product was purified

by column chromatography over silica gel eluting with petroleum ether/ethyl acetate (*v*/*v*, 7/1) to give the desired product as a white solid (111 mg, 90% yield): ^{1}H−NMR (400 MHz, DMSO-d_6) δ 9.14 (s, 1H), 8.63 (d, *J* = 8.4 Hz, 1H), 8.31 (d, *J* = 8.4 Hz, 1H), 8.14 (d, *J* = 7.6 Hz, 2H), 7.88 (d, *J* = 8.4 Hz, 1H), 7.56 (d, *J* = 6.9 Hz, 1H), 7.53–7.49 (m, 3H), 7.45 (s, 3H), 7.24 (d, *J* = 8.4 Hz, 1H). ^{13}C−NMR (100 MHz, DMSO-d_6) δ 152.07, 151.57, 151.22, 148.67, 148.09, 146.97, 134.01, 133.55, 131.23, 130.85, 130.74, 130.34, 129.32, 128.06, 127.63, 121.24, 118.91, 110.18. HRMS (ESI) calcd for $[M+H]^+$ $C_{20}H_{12}N_3O_9S$: 470.0294, found: 470.0286.

UV-Vis absorption spectroscopy and fluorescence spectra

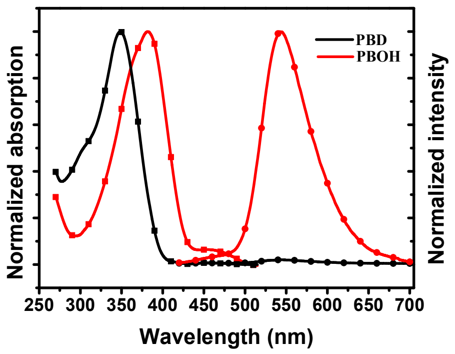

Figure S1. The absorption (■) and emission (●) spectra of **PBOH** and **PBD** in EtOH-PBS buffer (10 mM, pH = 7.4, 4:6, *v*/*v*).

Time-dependent fluorescence intensity of PBD with Cys and Hcy

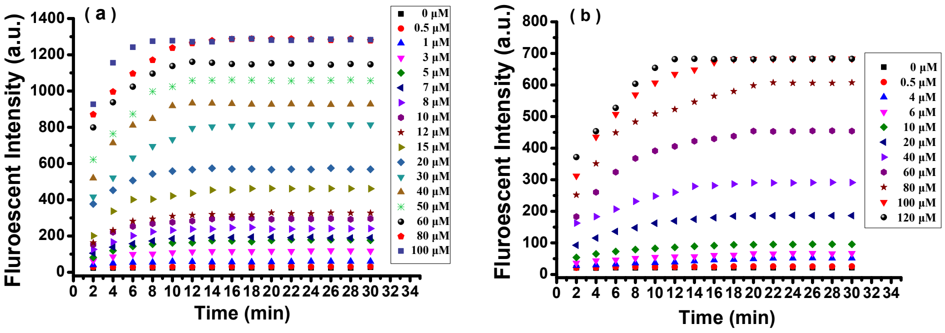

Figure S2. Time-dependent fluorescence intensity changes of **PBD** (10 μM) at 540 nm upon addition of varied concentrations of Cys (**a**) and Hcy (**b**) in EtOH-PBS buffer (10 mM, pH 7.4, 4:6, *v*/*v*) at room temperature.

Quantum yield calculation

Quantum yields were determined using *N*-ethyl-4-ethylamino-1, 8-naphthalimide as a standard according to a published method [2,3]. The quantum yield was calculated according to the equation

$\Phi_{sample} = \Phi_{standard} \times (A_{standard}F_{sample}/A_{sample}F_{standard})$

where Φ is the quantum yield, $\Phi_{standard} = 0.74$ in EtOH, F_{sample} and $F_{standard}$ are the integrated fluorescence intensities of the sample and the standard at the excitation wavelength, and A_{sample} and $A_{standard}$ are the optical densities of the sample and the standard, respectively.

Quantum yield of **PBOH**: $\Phi = 0.60$.

Quantum yield of **PBD**: $\Phi = 0.011$.

Effect of pH on fluorescence detection of biothiols

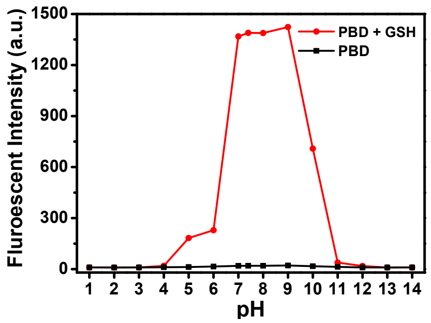

Figure S3. Fluorescence responses of PBD (10 μM) in the absence and presence of 100 μM GSH in 10 mM PBS buffer solutions containing 40% EtOH as a co-solvent at different pHs.

Calculation of detection limit

The detection limit was calculated according to the method used in the previous literature [4-6]. The fluorescence emission intensity (540 nm) was plotted versus the concentrations of biothiols. The detection limit was calculated using the equation

$$\text{Detection limit} = 3\,\sigma / k$$

where σ is the standard deviation of blank measurement and k is the slope between the fluorescence emission intensity versus GSH concentration. The detection limit was calculated to be 14.5 nM for GSH. Similarly, the detection limit was calculated to be 17.5 nM for Cys and 80.0 nM for Hcy (Figure S4), respectively.

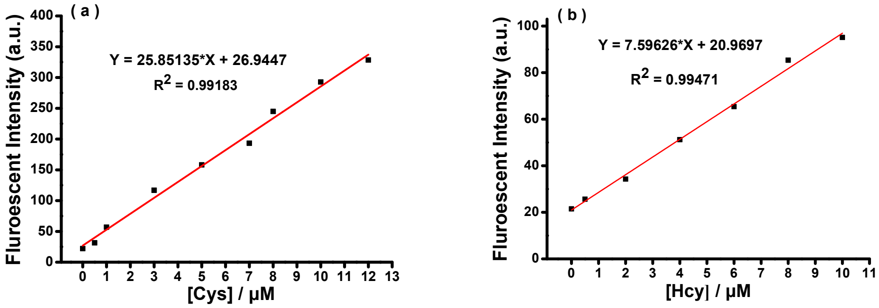

Figure S4. The fluorescence intensity of **PBD** (10 μM) at 540 nm was linearly related to the concentration of Cys (**a**) and Hcy (**b**).

Dynamic studies of PBD with biothiols

Pseudo-first-order rate constant [7,8].

The rate constant was determined according to the equation

$$\mathrm{Ln}\,[(F_{max}-F)/F_{max}] = -kt$$

where F and F_{max} are the fluorescence intensity at time t and the time after completion, respectively. The constant k obtained is shown in Figure S5. The values of rate constants of GSH, Cys, and Hcy were 0.25 min^{-1}, 0.26 min^{-1}, and 0.20 min^{-1}, respectively.

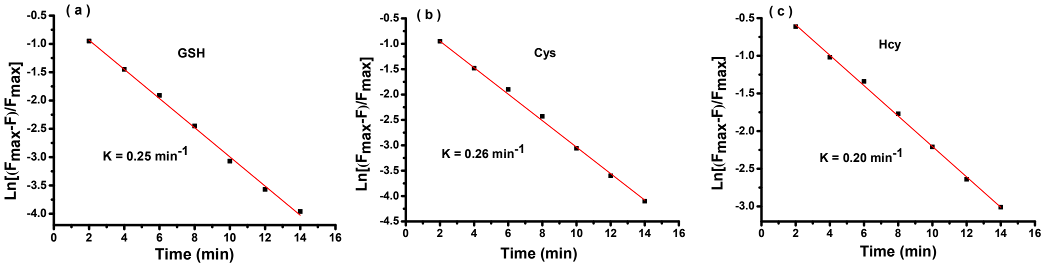

Figure S5. Pseudo-first-order rate figures of 10 μM **PBD** in the presence of 50 μM GSH (**a**), Cys (**b**), and Hcy (**c**) in PBS (10 mM, pH 7.4) containing 40% C_2H_5OH, λ_{ex} = 405 nm, λ_{em} = 540 nm.

Fluorescence response of PBOH, PBD with GSH and PBD

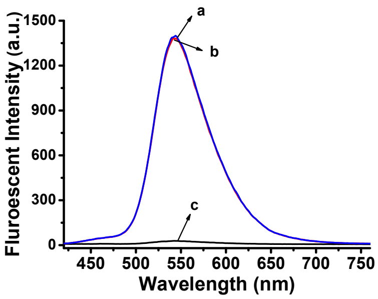

Figure S6. Fluorescence response of 10 μM **PBOH** (**a**), 10 μM **PBD** with 100 μM GSH (**b**), and 10 μM **PBD** (**c**) in EtOH-PBS buffer (10 mM, pH 7.4, 4:6, *v*/*v*).

Cytotoxicity measurements of PBD

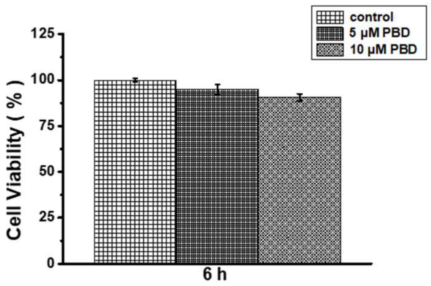

Figure S7. Cytotoxicity measurements of PBD. HeLa cells were treated with 5 μM and 10 μM PBD for 6 h. Then, CCK8 assay was used to measure cell viability.

NMR and HRMS data

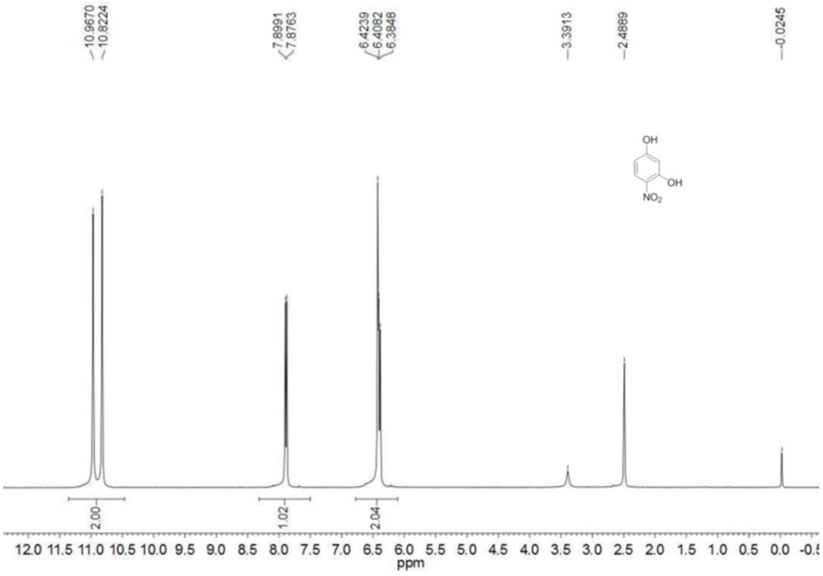

Figure S8. ^{1}H-NMR spectrum of compound 1 in DMSO-d_6.

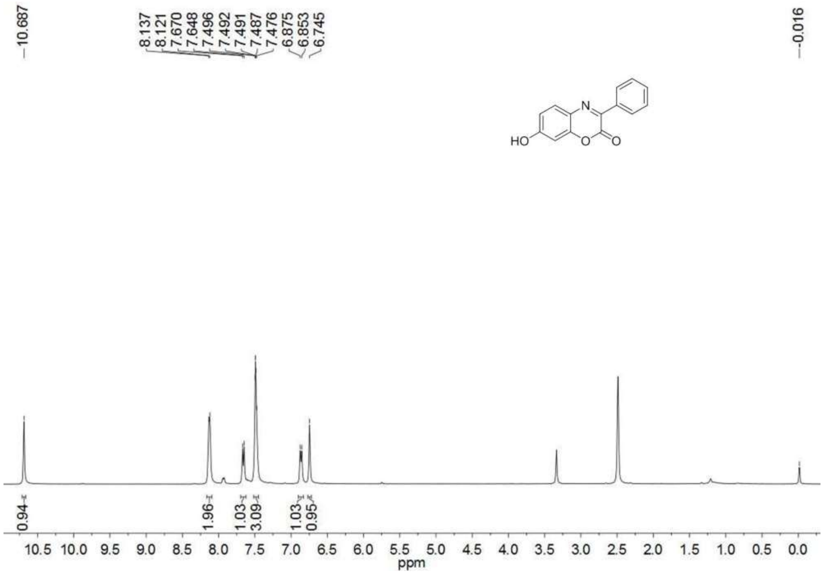

Figure S9. ^{1}H-NMR spectrum of **PBOH** in DMSO-d_6.

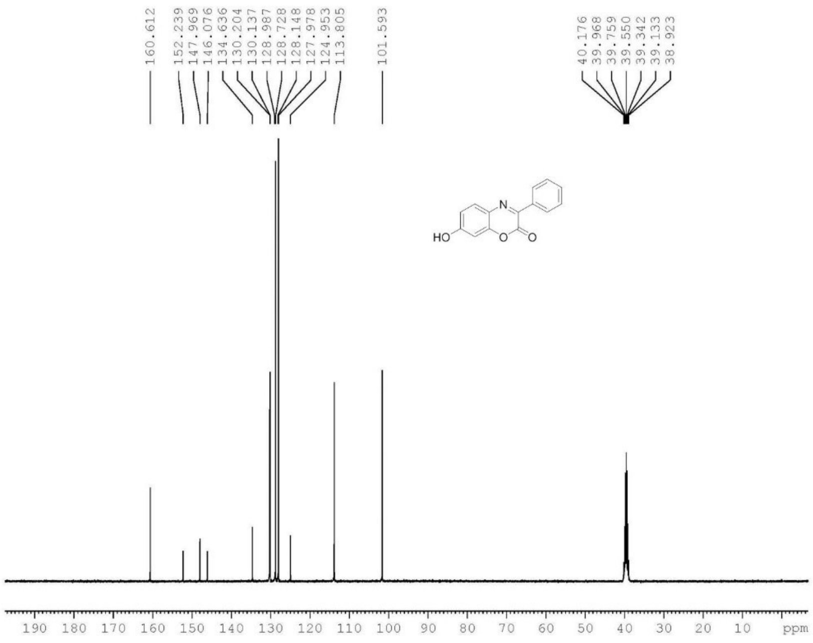

Figure S10. ^{13}C-NMR spectrum of **PBOH** in DMSO-d_6.

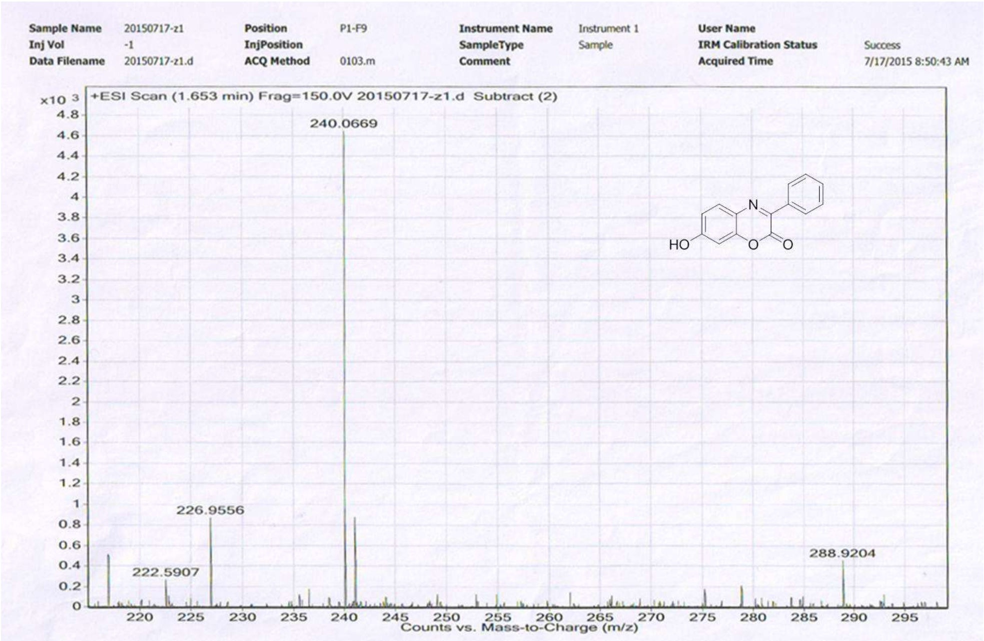

Figure S11. HRMS spectrum of **PBOH**.

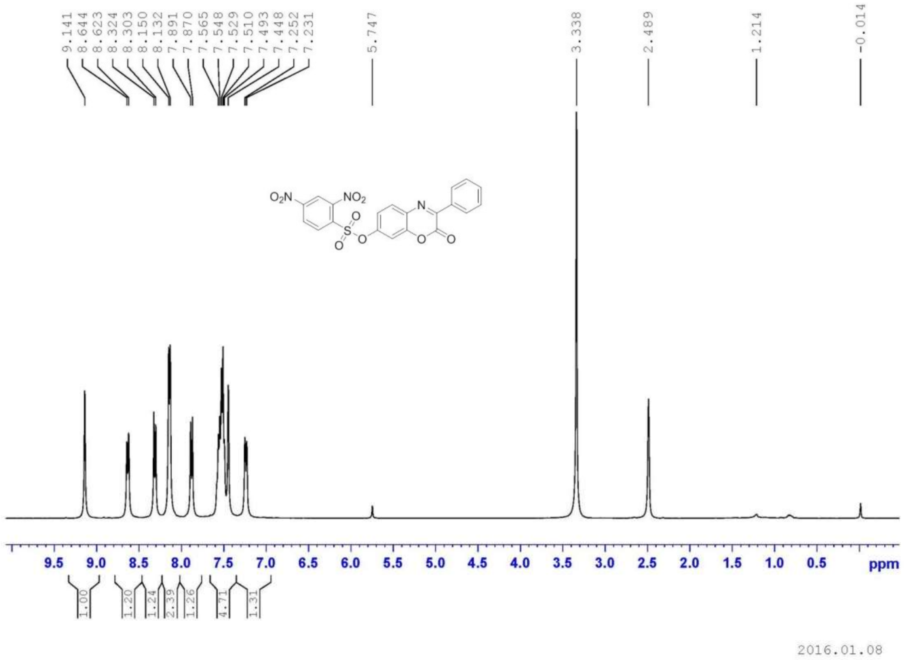

Figure S12. ^{1}H-NMR spectrum of **PBD** in DMSO-d_6.

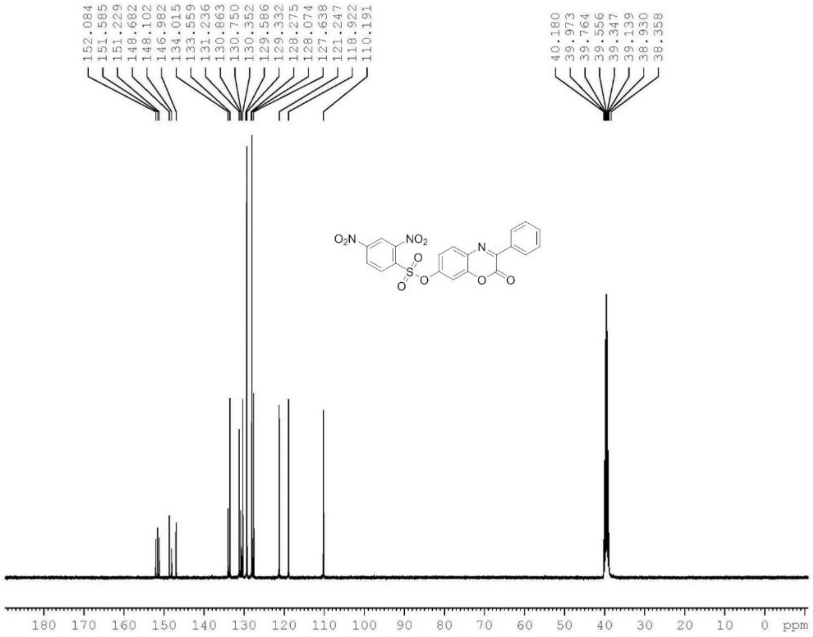

Figure S13. ^{13}C-NMR spectrum of **PBD** in DMSO-d_6.

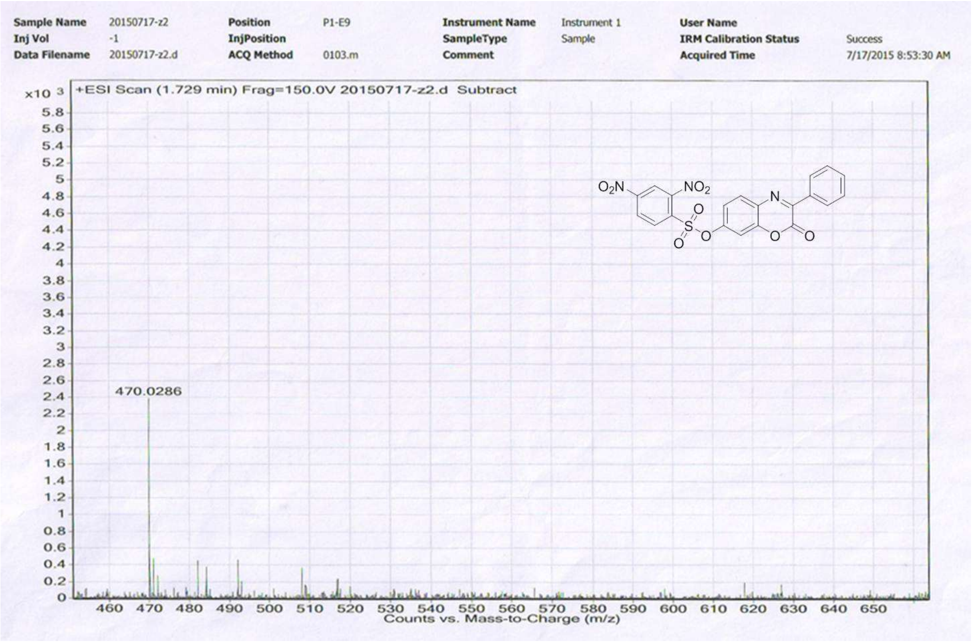

Figure S14. HRMS spectrum of **PBD.**

References

[1] Alexiou, M.S.; Tychopoulos, V.; horbanian, S.; Tyman, J.H.; Brown, R.G.; Brittain, P.I. The UV-Visible Absorption and Fluorescence of Some Substituted 1,8-Naphthalimides and Naphthalic Anhydrides. *J Chem.Informatics*. **1990**, *5*, 837–842.

[2] Jiang, W.; Fu, Q.; Fan, H.; Ho, J.; Wang, W. Alternate - Site Enzyme Promiscuity. *Angew. Chem. Int. Ed. Engl*. **2007**, *119*, 8597–8600.

[3] An, R.B.; Zhang, D.T.; Chen, Y.; Cui, Y.Z. A "turn-on" fluorescent and colorimetric sensor for selective detection of Cu^{2+} in aqueous media and living cells. *Sens. Actuators: B*. **2016**, *222*, 48–54.

[4] Wang, Z.; Han, D.M.; Jia, W.P.; Zhou, Q.Z.; Deng, W.P. Reaction-Based Fluorescent Probe for Selective Discrimination of Thiophenols over Aliphaticthiols and Its Application in Water Samples. *Anal. Chem*. **2012**, *84*, 4915–4921.

[5] Yu, D.; Huang, F.; Ding, S.; Feng, G. Near-Infrared Fluorescent Probe for Detection of Thiophenols in Water Samples and Living Cells. *Anal. Chem*. **2014**, *86*, 8835–8841.

[6] Kand, D.; Mandal, P.S.; Datar, A.; Talukdar, P. Iminocoumarin based fluorophores: Indispensable scaffolds for rapid, selective and sensitive detection of thiophenol. *Dyes and Pigments*. **2014**, *106*, 25–31.

[7] Dale, T.J.; Rebek, J. Fluorescent sensors for organophosphorus nerve agent mimics. *J. Am. Chem. Soc*. **2016**, *128*, 4500–4501.

[8] Ji, W.; Ji, Y.; Jin, Q.; Tong, Q.; Tang, X. Heavy atom quenched coumarin probes for sensitive and selective detection of biothiols in living cells. *Analyst*. **2015**, *140*, 4379–4383.
